# PathogenFinder - Distinguishing Friend from Foe Using Bacterial Whole Genome Sequence Data

**DOI:** 10.1371/journal.pone.0077302

**Published:** 2013-10-28

**Authors:** Salvatore Cosentino, Mette Voldby Larsen, Frank Møller Aarestrup, Ole Lund

**Affiliations:** 1 Center for Biological Sequence Analysis, Department of Systems Biology, Technical University of Denmark, Kgs. Lyngby, Denmark; 2 National Food Institute, Technical University of Denmark, Kgs. Lyngby, Denmark; University of Hawaii Manoa, United States of America

## Abstract

Although the majority of bacteria are harmless or even beneficial to their host, others are highly virulent and can cause serious diseases, and even death. Due to the constantly decreasing cost of high-throughput sequencing there are now many completely sequenced genomes available from both human pathogenic and innocuous strains. The data can be used to identify gene families that correlate with pathogenicity and to develop tools to predict the pathogenicity of newly sequenced strains, investigations that previously were mainly done by means of more expensive and time consuming experimental approaches. We describe *PathogenFinder* (http://cge.cbs.dtu.dk/services/PathogenFinder/), a web-server for the prediction of bacterial pathogenicity by analysing the input proteome, genome, or raw reads provided by the user. The method relies on groups of proteins, created without regard to their annotated function or known involvement in pathogenicity. The method has been built to work with all taxonomic groups of bacteria and using the entire training-set, achieved an accuracy of 88.6% on an independent test-set, by correctly classifying 398 out of 449 completely sequenced bacteria. The approach here proposed is not biased on sets of genes known to be associated with pathogenicity, thus the approach could aid the discovery of novel pathogenicity factors. Furthermore the pathogenicity prediction web-server could be used to isolate the potential pathogenic features of both known and unknown strains.

## Introduction

Every year more than 15 millions deaths are the direct cause of infectious diseases, many of which are due to bacterial infections. Each year an estimated 1.3 million people die of tuberculosis and 0.2 millions of pertussis, while diarrhoea accounts for more than 2.5 millions deaths, and is one of the leading causes of death in worldwide [1]. But not all bacteria are dangerous and many of them are innocuous or even beneficial to human. The gut of a healthy adult human contains thousand of different microbial species, many of which are beneficial to their host, providing functions for nutrition and development, and regulating the immune response [2], [3]. Nevertheless some bacterial species, like *Escherichia coli*, also include extremely deadly strains, causing for example diarrhoea, urinary tract infections, septicaemia etc. Thus identifying pathogenic strains and understanding the biological mechanisms that cause them to become pathogenic is highly important to perform timely interventions and design control strategies, including interventions such as restrictions on contaminated food products, isolation of patients, correct treatment and development of targeted vaccines.

Ever since the 1880s the pathogenicity of bacteria have been assessed using Koch’s postulates, for human pathogens using animal models. During the last 2 decades many discoveries have shown that Koch’s postulates are not enough to decide if a given bacteria is pathogenic or not. The existence of diseases caused by bacteria that cannot grow in pure culture medium [4], [5], the discovery of polymicrobial diseases [6], the role of metagenomic microbiota in chronic diseases [7], and last but not least, the discovery of Horizontal Gene Transfer (HGT) responsible for the swapping of genetic material between bacteria [8] (regardless the pathogenicity), are all cases in which the postulates have short-comings. Already during his work with *Vibrio cholerae* Robert Koch himself discovered the shortcomings of animal models for correctly identifying human-specific pathogens. Thus, the use of animal models is not always reliable in defining if a given bacteria is human pathogenic. Moreover, assessing the pathogenicity by means of animal models or epidemiological studies is both time-consuming and expensive.

Among the molecular features that a bacterium needs to infect and survive inside its host [9] are exotoxins, endotoxins, two components systems [10], adherence factors, secretion systems (I to IV type) [11], through which bacteria can inject their toxins into its hosts cells [12]. Plasmids, secretion systems, and antibiotic resistance genes are commonly present in both commensal and pathogenic strains, while toxins are usually only present in pathogenic strains. There are many databases available containing genes encoding toxins and virulence factors along with other genes traditionally associated with pathogenicity [13], [14].

One of the ways to classify a bacterium as human pathogenic using bioinformatics was (and still sometimes is) to look for some of these features in the genome of the isolate under investigation. Unluckily this approach is not always reliable, partly because of HGT, which causes these features to be exchanged among pathogenic and innocuous strains of the same [15] [16] or different species, an exchange which has been proved by the high amount of these features found in genomic islands [17]. Aside from the features directly associated to pathogenicity, there are also virulence “lifestyle” genes, important for the bacteria to survive inside the host and evade its immune system response [18] [19], and genes that are, for example, needed to activate other genes, which are important in the processes of pathogenesis, even though they do not directly determine virulence. All the issues related to the prediction of bacterial pathogenicity based on phylogeny has caused researchers to look for different solutions.

The development of whole genome sequencing may open novel ways of predicting pathogenicity in bacterial species. In 1995 the genomes of *Mycoplasma genitalium* and Haemophilus influenzae [20], [21] were completely sequenced, and scientists started considering the possibility of studying the pathogenesis of bacteria based on their genome sequences [22]. This was the start of a revolution that has been continuing during the last decade with the advent of Second-Generation or Next-Generation Sequencing (NGS), leading to a continuous decrease in sequencing costs and a fast development of sequencing technologies. At present, many different high-throughput sequencing systems are available [23]–[25] and the number of completely sequenced bacteria amount to almost 2,400 including more than 1,800 that have been submitted to the International Nucleotide Sequence Database Collaboration (INSDC) (www.genomesonline.org, May 2013).

A few methods have been proposed which make use of Support Vector Machines (SVM), BLAST or other bioinformatics tools to search for pathogenic features [26], [27] or predict bacterial pathogenicity [28] by searching in pre-computed databases of genes associated with pathogens. One shared aspect among these methods is the fact that they restrict their search to well known pathogenic features, missing out on the information that may be contained in the many genes with unknown function. Furthermore, the methods ignore genes that could be shared and specific among non-pathogenic organisms. When bacteria become pathogenic through HGT their lifestyle change and some of the genes may be inactivated or even lost to adapt to the new lifestyle [29], [30]. These genes are still present in non-pathogenic bacteria and hence could be used, together with the genes associated to pathogenicity, to separate dangerous bacteria from harmless ones.

As an alternative to the above mentioned prediction methods, we here developed a novel approach, building on a previous study [31]. In this study we selected groups of genes which are frequently found either in human pathogenic bacteria or in the innocuous ones, and show that this is more effective than using global similarity. Since we did not make any pre-assumption on the genes contained in our training-sets, we are able to identify new proteins associated to pathogenicity and also features shared among non-pathogenic bacteria. Moreover, our hypothesis-free approach gave us the chance to build, together with a phylogenetic-independent model using all the organisms we have, more specific models grouping organisms at different taxonomic ranks to improve the predictions in species like *E. coli*, in which the high amount of shared genes among pathogenic and commensal strains makes it particularly difficult to predict. In this study the original approach [31] was, furthermore, extended from 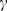-proteobacteria to all species and extended to not only give a prediction, but also identify which genes predicted to be most significantly associated with (or important for) pathogenicity or non-pathogenicity. Thus, the method will not only provide a prediction of pathogenicity, but may also be useful for identifying novel putative pathogenicity genes, supporting further functional genomic studies.

The predictor has been implemented as a free to use web-service, called *PathogenFinder*, to which users can upload raw reads, obtained from different NGS sequencing platforms, as well as assembled genomes, and obtain a fast estimation of the pathogenic potential of the bacteria they are studying, as well as the identification of potentially pathogenic genes. *PathogenFinder* could be helpful in situations of possible bacterial outbreaks, in which a fast analysis of the unknown strain is important to save lives, and follows the direction modern clinical microbiology [32] and global epidemiology [33] are taking driven by the revolution brought by high throughput DNA sequencing technologies.

## Results and Discussion

### Overview on the Created Models

In this work we developed a method for predicting the pathogenicity of novel bacteria. We did this by comparing the proteins of the strain under investigation to a protein family database (PFDB) composed of groups of proteins (protein families or PFs) that were either associated with pathogenic or non-pathogenic organisms. In the creation of the PFDB we used 885 complete bacterial genomes (Table S1), 372 of which were tagged as human pathogens and 513 as non-pathogens.

All the proteins encoded by the bacterial genomes were initially clustered, and significant clusters, in which the majority of the proteins originated from either pathogens or non-pathogens, were identified. The PFs were accordingly tagged as pathogenic or non-pathogenic and a weight (Z-score) was calculated for each of them (see Materials and Methods for further details). Eight models were built using bacteria belonging to the same phylum or class as training data (Table 1). These models are named TM-*taxname*, where *taxname* is the phylum or class (e.g., bacteroidetes) of the organisms in the training data. Two other models created were: the whole-data model (WDM), which was trained using all the 885 bacteria in our training-set; the complement model (COMPL), which was trained using the organisms belonging to classes and/or phyla for which we had either only pathogenic or non-pathogenic strains and for which it was hence not possible to create specific models (Table S1).

**Table 1:** Training, test data and model parameters.

	Training Set			Test Set			Model Parameters			
Model Name	Pathogenic	Non-pathogenic	Total	Pathogenic	Non-pathogenic	Total	MinORG	LT	HT	Zthr
TM-Alphaproteobacteria	29	60	89	11	28	39	2	0.15	0.6	10.43
TM-Betaproteobacteria	26	26	52	10	22	32	2	0.3	0.9	0.55
TM-Epsilonproteobacteria	17	5	22	16	2	18	2	0.4	1.0	−9.31
TM-Gammaproteobacteria	122	97	219	33	50	83	2	0.2	0.85	25.37
TM-Actinobacteria	27	44	71	24	36	60	2	0.0	1.0	−3.22
TM-Bacteroidetes	7	12	19	5	24	29	2	0.35	0.6	1.68
TM-Firmicutes	98	87	185	34	83	117	3	0.0	1.0	−2.85
TM-Tenericutes	6	8	14	5	9	14	2	0.0	1.0	−1.59
COMPL	40	174	214	17	40	57	2	0.0	1.0	−1.78
WDM	372	513	885	155	294	449	2	0.0	1.0	3.0

Training, test data and model parameters. The last 3 columns show the MinORG, LT and HT parameters used to create the pathogenicity families and build the model for each of the 10 models. *Zthr* is a threshold value, calculated for each model at the cross validation phase, which is used, given the final prediction score, to decide if the input organisms will be predicted as pathogenic or non-pathogenic. The parameters for each model are chosen after 5-fold cross-validation tests.

Given a query organism, based on the number and kind of PFs that the proteins of the query organism are similar to, a prediction on whether it is human pathogenic or non-pathogenic is performed. The predictor has been implemented as a free to use web-server called *PathogenFinder*, to which a user can upload either the raw reads or the complete or draft genome of the organism they want to assess the pathogenicity of. One of the 10 built models can be selected for the prediction, and if the user does not know which class or phylum the organism belongs to, the web-server will identify it automatically by predicting 16S genes, using *RNAmmer* [34], and accordingly select the appropriate model to be used for the prediction.Both the set of matches used for the prediction and the raw matches from *PathogenFinder* are downloadable. The latter is particularly useful, since it contains more information about pathogenicity than the standard server output, and could hence be used for a more detailed analysis of the pathogenicity features of the organisms under investigation.

### Performance on Five-Fold Cross Validation and Independent Test Data

The TM models were tested using only organisms belonging to the specific phylum/class, while in the case of the WDM model the whole independent data-set was used for the test.

Table 2 shows the performance of the ten models as obtained by 5-fold cross validation (CV) (column 2) and on independent test-sets of organisms from the same taxonomic group (column 3). As can be seen for the Tenericutes and Bacteroidetes phyla, the performances were very poor when compared to the MCC obtained in the CV tests. This is likely to be caused by the models being built using a small number of organisms (Table 1). For instance, the TM-Tenericutes model was trained on only 14 isolates. Furthermore, it was tested on a set of organisms from species that were not present in the training-set.

**Table 2:** MCC on cross validation and independent test-set.

Organism subset	5-fold CV	TM or COMPL	WDM
All Bacteria	0.847	0.736^3^	0.758
*α*-proteobacteria	0.949	0.886	0.873
*β*-proteobacteria	0.923	0.855	0.79
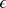 -proteobacteria	0.741	0.686	1.0
*γ*-proteobacteria	0.825	0.666	0.661
Actinobacteria	0.681	0.816	0.826
Bacteroidetes	0.889	0.535	0.383
Firmicutes	0.915	0.756	0.785
Tenericutes	0.866	−0.344	0.0
Remaining Organisms^1^	0.940	0.793	0.877^2^

Column 2, the MCC obtained in the 5-fold cross validation (CV) by each of the 10 models. Column 3, the MCC of the individual TM models and the COMPL model (last line) when tested on independent test data from the corresponding phyla/classis. Column 4, the MCC of the WDM model when tested on independent test data from specific phyla/classis.

1 Organisms of phylum/class for which no TM model is available were tested using COMPL model. COMPL was trained on all organisms from classes or phyla for which only either pathogenic or non-pathogenic strains were available.

2 MCC for WDM on the same test-set used for COMPL.

3 Overall MCC for all the TM models and the COMPL model.

To compare the performance of the WDM model to those of the TM and COMPL models, we examined the MCC of the WDM on the same test-sets used for the other models (column 4 in Table 2).

For example, to examine the performance of the WDM in predicting the pathogenicity of Firmicutes bacteria, we tested it with the same organisms used to assess the accuracy of the TM-Firmicutes model.

The MCC obtained by the WDM (0.758) on all bacteria was higher than the overall accuracy of all the TM models and COMPL model combined (0.736). Nonetheless, the TM models performed better for bacteroidetes, 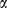, 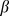, and 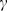-proteobacteria, even though for the latter the difference from the WDM was not significant. The remaining TM models and the COMPL model had lower MCC than the WDM for the same organisms.

### Performance on Draft Genomes and *Escherichia coli*

The models ability in predicting the pathogenicity of an isolate as based on a draft genome was tested using 259 sets of illumina raw reads from 6 different species. While in the case of *Campylobacter jejuni*, *Klebsiella pneumoniae* and *Staphylococcus aureus* (57 isolates in total) all the predictions were correct, the results were not satisfactory for Enterococci and *E. coli*. Of 50 *Enterococcus faecalis* and 49 *Enterococcus faecium* from healthy Danish pigs, all isolates were predicted as pathogenic. Our training-set only contained a single pathogenic *E. faecalis* and no *E. faecium*, which may explain these results.

The WDM as well as the TM-Gammaproteobacteria models predicted the 10 *E. coli* strains in the test-set as pathogenic, although 4 strains were annotated as non-pathogenic. A similar situation was observed for the 103 *E. coli* draft genomes. Accordingly, we decided to create a model only for the *Enterobacteriaceae* family, using the organisms in our training-set. The resulting model correctly predicted 1 of 4 non-pathogenic *E. coli* achieving an MCC of 0.41, but all draft genomes were still predicted as pathogenic. The model also showed improvements in predicting other *Enterobacteriaceae*, with an MCC of 0.675, while WDM and TM-Gammaproteobacteria had an MCC of 0.519 and 0.617, respectively.

To improve the predictions for *E. coli* further, we decided to create 2 special models. These models were called *ecoli_boost* and *enterobac_boost*, and they were trained on a set that was enriched with 14 extra non-pathogenic *E. coli* strains downloaded from the National Center for Biotechnology Information (NCBI) (Table S2). These two models had a noticeably improvement on both CG test-sets and on the 103 assembled *E. coli* isolates, on which MCC was 0.346 (Acc = 67%) and 0.360 (Acc = 68%) for *enterobac_boost* and *ecoli_boost*, respectively. The lists of organisms used to train the *enterobac_boost* and *ecoli_boost* models, together with more details on the results on *E. coli* can be seen in Table S2.

### Comparison to other Prediction Methods

Presently, the literature describes two main approaches for predicting the human pathogenicity of bacteria based on whole genome sequencing data: the first, proposed by Andreatta et al. [31], is able to predict the pathogenicity of 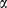-proteobacteria, and it was from this study we borrowed the concept of PFs; the second method, developed by Iraola et al. [28], uses SVM [35], and can predict the pathogenicity of all types of bacteria. In this method the authors selected 120 genes associated to pathogenicity from 600 complete genomes using SVM, and built a prediction model based on the selected genes.

To compare our method to the one proposed by Andreatta et al., we built a model using the same set of 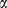-proteobacteria organisms (155) and the same parameters (MinOrg, HT, LT) used by Andreatta et al. The key differences between our method and the one by Andreatta et al. are: 1) we used CD-HIT instead of BLAST in both the protein clustering and prediction phases; 2) we used Equation 3 to filter the significant matches of the query sequences, while Andreatta et al. filtered based on a BLAST e-value threshold; 3) We compute the final predictions using the Z-scores, while Andreatta et al. counted the number of pathogenic and non-pathogenic families matched. The obtained model was tested on the same independent set used by Andreatta et al. This set included 24 organisms (14 pathogenic), and our model was able to correctly classify 23 organisms (95.8%). This is equivalent to an MCC of 0.92, while Andreatta’s MCC was 0.837. The one organism that our method was not able to correctly classify, is *Salmonella enterica* Serovar Gallinarum str. 287/91 [GenBank:30689], which is pathogenic for poultry, but not known to be for humans. The pathogenicity of this organism is restricted to chicken although it shares a high quantity of genomic features associated to pathogenicity with its human pathogenic ancestor *Salmonella Enteriditis* [36]. It is likely that these features mislead the prediction model, since also the method by Andreatta et al. wrongly classified this *S. enterica* strain.

To compare our method to the predictor proposed by Iraola et al., we used the test-set they used for their blind test evaluation. The test-set, originally composed of 233 organisms, contained 5 strains, which were excluded from the comparison, since they were also present in our training-set. Overall, for the comparison, we had a test-set composed of 228 organisms, 192 of which are tagged as human pathogens and the remaining 36 as non-pathogens.

*PathogenFinder* achieved an overall MCC of 0.67 for the taxonomy models and 0.65 for the WDM model. Both results are higher than the MCC of 0.6 obtained by the method proposed by Iraola et al. Table S3 contains a detailed description of the comparison, including the organisms used and the corresponding predictions from both methods.

### PFDB Analysis and Biological Interpretation

For each created model, an analysis of its PFDB was performed and its PFs ranked based on their Z-scores. The scores above 0 are associated with pathogenic PFs, while those below 0 are associated with non-pathogenic PFs. No protein function analysis was done prior to the models creation, making the approach unbiased on the genomic content of the organisms, regardless of their pathogenicity. In this paragraph we describe the analysis of the PFs of the TM-Gammaprobacteria and WDM.

The analysis of the PFDB of TM-Gammaproteobacteria model showed that the high ranked pathogenic families (Table 3) contained proteins well known to be associated to pathogenicity. The family at rank 1 and 3 contained N-acetylmannosamine kinase, which is a key enzyme in sialic acid synthesis and sialic acid transport proteins. Sialic acid is important for virulence and is believed to help the microbes to disguise themselves as host cells in order to elude the host’s immune system response [37]. Fimbrial proteins (rank 2) are important for bacterial adherence [38]. At rank 10 we found cytochrome b_562_ proteins that help bacteria to survive and grow in conditions of poor oxygen [39]. Other high-ranked families contained proteins associated with secretion systems (II and III) and antibiotic resistance.

**Table 3:** Top 10 ranking pathogenic protein families and annotated functions of their proteins for TM-Gammaproteobacteria model.

RANK	Z-score	P	N	Function
1	9.134	77	8	N-acetylmannosamine kinase (TCS)
2	8.500	49	0	Fimbrial proteins
3	8.170	62	6	Sialic Acid Transporter
4	8.158	53	3	Transposition helper protein
5	8.023	62	7	Acetyltransferase, type III secretion proteins
6	8.023	62	7	Macrolide-specific efflux, membrane protein
7	8.023	62	7	Type II secretion proteins
8	7.922	69	10	Unknown function, possible membrane proteins
9	7.906	60	7	Unknown function
10	7.855	53	4	Cythochrome b_562_

P and N columns contain the number of pathogenic and non-pathogenic organisms in the protein family respectively.

An interesting finding, which was also found in [31], was the presence of families containing proteins with unknown functions associated with pathogenicity. This finding suggests that those proteins with unknown function might have important roles in the bacterial pathogenesis and could form the basis for further functional studies improving our understanding of bacterial pathogenicity. Proteins with unknown functions were also identified as associated with non-pathogenic PFs (Table 4).

**Table 4:** Top 10 ranking non-pathogenic protein families and annotated functions of their proteins for TM-Gammaproteobacteria model.

RANK	Z-score	P	N	Function
1	−6.52	3	34	Protein-L-isoaspartate
2	−6.44	2	31	ThiJ/PfpI domain protein
3	−6.43	6	40	Anthranilate synthase component I
4	−5.98	6	36	8-amino-7-oxononanoate synthase
5	−5.92	5	34	Unknown function, putative transcriptional regulator
6	−5.82	0	21	Adenosylmethionine decarboxylase
7	−5.81	8	39	Unknown function
8	−5.80	2	26	Unknown function, probable condensation protein
9	−5.68	0	20	Nitrite transporter
10	−5.62	1	22	Glucose-galactose transporter

P and N columns contain the number of pathogenic and non-pathogenic organisms in the protein family respectively.

The analysis of the PDBF of the WDM enabled us to see if proteins involved (or not involved) in pathogenesis belong to organisms of different taxonomy, and at the same time gave us an insight on how proteins are conserved along the different phyla. Again, we found that the top ranked families associated to pathogenicity (Table 5) contained also proteins with unknown function.

**Table 5:** Top 10 ranking pathogenic protein families and annotated functions of their proteins for the WDM model.

RANK	Z-score	P	N	Function
1	10.18	38	0	Borrelia Plasmid partition proteins
2	9.49	33	0	TCS associated genes, unknown functions
3	9.19	31	0	Lipoate-protein ligase, lipoate metabolism associated proteins
4	9.19	31	0	Unknown functions, flavin oxidoreductase
5	9.04	30	0	Exfoliative toxin A
6	8.89	29	0	Pili assembly proteins, Motility, Secretion Systems
7	8.89	30	0	Unknown function, shikimate kinase
8	8.89	29	0	Pili assembly proteins, Motility, Secretion Systems
9	8.74	28	0	Multiple antibiotic resistance (MarR) family proteins
10	8.74	28	0	Mutarotase Yjht (sialic acid mutarotation), unknown functions

P and N columns contain the number of pathogenic and non-pathogenic organisms in the protein family respectively.

The highest ranked PF contained proteins encoded by plasmids from different pathogenic *Borrelia* species (mainly *Burgdorferi*), which are involved in pathogenesis [40], [41]. The family ranked 3*^rd^* contained proteins associated with lipoate metabolism. The acquisition and use of lipoate by pathogens affect their virulence and the pathogenesis of the diseases they cause [42]. Among the toxins found were: exofiliative toxin A (family-rank 5) in *Staphylococcus aureus* strains, causative of Staphylococcal scalded skin syndrome [43], [44]; streptolysin (O and S), mainly found in *Streptococcus* pathogenic species [45]; hemolysin (II, III, 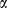 and 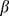 types) found in PFs mainly composed of 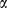-proteobacteria [46], [47] and firmicutes organisms [48]; shiga toxin, a common pathogenicity factor in many virulent *E. coli* strains [49]; dermonecrotic toxin (DNT), one of the main virulence factors in many *Bordetella* species [50](pertusiss in human), but at the same time present in plant pathogenic organisms like *Erwinia amylovora* [51] and *Erwinia pyrifoliae* [52]. The fact that we could find PFs containing DNT tagged as pathogens and others tagged as non-pathogenic (like the one containing DNT for *E. amylovora* and *E. pyrifoliae*) is an example of the ability of our clustering method to associate a given protein (a toxin in this case) to human pathogenicity as well as non-pathogenicity depending on the organism in which it is found.

Another example through which we could see the discriminative power of our PFs, was in associating pathogenicity to the different secretion system types proteins (SST1–SST6). For SST3 we identified 284 protein families, 147 of which were tagged as pathogenic. The pathogenic PFs were composed of human pathogenic 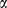-proteobacteria strains, while the non pathogenic PFs contained plant pathogenic organisms from proteobacteria genera like *Xanthomonas*, *Agrobacter* and *Erwinia*, which use SST3 (and other secretion systems) to infect the hosts cells of plants [53], [54].

The protein families with high rank associated with non-pathogenicity (Table 6) were usually composed of proteins present in bacteria living in hot springs, lake surfaces or deep in the sea, and the functions are associated to their ability to survive under those extreme environmental conditions. Among those proteins are Rubrerythrin, found in anaerobic sulphate-reducing bacteria like *Geobacter* and *Desulfivibrio* [55]. When the PFs were not composed of proteins from environmental bacteria, they contained mainly probiotics or plant pathogens. It is important to note that since the WDM model was created with HT and LT parameters with values of 1.0 and 0 respectively, we only have PFs composed of proteins from either only pathogenic organisms or only non-pathogenic organisms.

**Table 6:** Top 10 ranking non-pathogenic protein families and annotated functions of their proteins for the WDM model.

RANK	Z-score	P	N	Function
1	−6.68	0	63	tRNA proteins
2	−6.62	0	62	ABC transporter related proteins (for 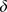 and 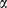 -proteobacteria)
3	−6.18	0	54	Rubrerythrin
4	−6.07	0	52	Rubrerythrin
5	−6.01	0	51	Iron-sulfur binding domain proteins
6	−6.01	0	51	Hydroxymethylglutaryl-CoA synthase
7	−5.95	0	50	Unknown function
8	−5.89	0	49	Unknown function
9	−5.83	0	48	Unknown function
10	−5.70	0	46	Sulfite reductase subunit

P and N columns contain the number of pathogenic and non-pathogenic organisms in the protein family respectively.

### Conclusions

There is an increasing need for fast identification of unknown bacteria with particular focus on the assessment of their potential pathogenicity. In this work we presented *PathogenFinder*, a web-server that by analysing the user-uploaded proteome can identify genomic features associated with both pathogenicity and non-pathogenicity. Given an input proteome the method quickly predicts its potential pathogenicity, making it a useful tool to be used together with other web-services developed for bacterial outbreak surveillance. Moreover, the possibility for the user to download the complete set of predicted pathogenicity features for the input organism makes *PathogenFinder* convenient for the analysis of pathogenic and harmless strains for microbiologists, epidemiologist and in general institutions studying bacterial pathogenesis.

One of the novel aspects in our approach is in the construction of the prediction models, which was carried out without any prior analysis of the proteins in our training-set, by just tagging our organisms as pathogenic or non-pathogenic and identifying protein families that were frequently found in pathogenic or non-pathogenic organisms.

It is important to notice that even though an isolate may have been obtained from a non-pathogenic environmental or animal or human related source it is not necessarily non-pathogenic. Such strain might in fact be highly pathogenic opportunistic pathogens. This naturally makes the creation of the optimal reference database difficult, but with increased number of isolates with well-defined meta data this is should still be doable.

We observed how *PathogenFinder* performs better than other pathogenicity prediction methods described in the literature, which usually rely on taxonomy and global sequence similarity with small sets of genes known to be associated with bacterial pathogenesis. We had less good results for species of the tenericutes phylum, and extra work need to be done to obtain statistically significant results for opportunistic strains (e.g. S. aureus) for which we could not tag any of our strains as non-pathogenic. The accuracy in predicting opportunistic bacteria could be improved by building specific models (e.g. at species level) as soon as new strains are available and there is a reasonable amount of both pathogenic and harmless strains. We have also shown how the prediction accuracy can be enhanced by increasing the number of organisms in the training-sets and/or making specific models at different taxonomic ranks, showing the example of *E. coli*, which is particularly difficult to predict because of the high similarity between commensal and pathogenic strains.

With the fast growing number of available bacterial complete genomes and with the increasing quality of the meta data we envision the possibility in the near future to build prediction models targeting only bacteria of a given genus or species, or even better, to build models to identify pathogenic features involved in specific diseases.

## Materials and Methods

### Training and Test Data

All available complete bacterial genomes (NCBI Genome Project, accessed on 10*^th^* Nov. 2010) were considered for the creation of the training-sets.

The pathogenicity information for the retrieved organisms were taken from NCBI genome project pages as described in Andreatta et al. [31], and for 885 of the 1,224 downloaded organisms, we were able to find pathogenicity information. The final complete training-set (Table S1) was composed of 513 organisms tagged as human non-pathogens and 372 tagged as human pathogens. For the human pathogenic organisms we checked for evidence in the literature.Opportunistic pathogens (e.g. from species like *Staphylococcus aureus* [56] or *Pseudomonas aeruginosa* [57]) were still tagged as pathogenic even though it has been shown that some of them can live inside the host without causing any disease, and their pathogenicity is sometimes related to the host’s health conditions.

From January 2012, NCBI removed pathogenicity information from its pages, redirecting the users to Genomes Online Database (GOLD) [58]. On 26*^th^* Feb. 2012 we queried GOLD for pathogenicity information about organisms that had been published after 5*^th^* Nov. 2010 (the date of the latest published bacteria in the training-set). We were able to extract pathogenicity information for 449 organisms, and subsequently retrieved the corresponding complete genomes and plasmids from NCBI based on the NCBI project ids.

The final test data (Table S1) was composed of 449 organisms, 294 of which were tagged as human non-pathogens and 155 as human pathogens.

### Protein Clustering

The model creation consisted of the following 2 main steps:

- Protein Clustering
- PFDB Creation

The initial idea for clustering the proteins was to use BLAST [59], but due to the size of our dataset (almost 3 million proteins), it would not have been computationally feasible. Instead, we used CD-HIT [60], which made it possible to cluster all the proteins in approximately 24 days using 2 3 Ghz dual-core CPUs in parallel and a 8 Gb of RAM.

The output from the program were 3 files containing respectively: 1) a list of cluster ids followed by the FASTA headers of the sequences composing the clusters; 2) a FASTA file containing all the clusters representative sequences; 3) a FASTA file containing all the solitary sequences that could not be included in any cluster.

### Protein Family Database (PFDB) Creation

Our prediction models are based on the concept of protein families as initially proposed in Adreatta et al. [31]. Protein families are groups of proteins with a certain degree of similarity. The PFs were created using a two-steps filtering of the clusters created using CD-HIT. To perform this filtering we used four parameters: MinORG, P*_ratio_*, LT and HT.

Let ORG be the number of organisms which have proteins in a given cluster *i*. We define MinORG as the minimum number of organisms that must have proteins in the *i* cluster for it to be considered significant. As such, MinORG is a lower threshold for the ORG value.

#### Equation 1

Ratio of human pathogenic organisms having proteins inside the cluster *i* on the total number of organisms having proteins in *i*. Newton’s Second Law
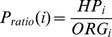
(1)


P*_ratio_* (Equation 1) is the ratio of the number of pathogen organisms having proteins in the *i* cluster (HP*_i_*) on the total number of organisms in *i* (ORG*_i_*). LT and HT are thresholds for the P*_ratio_* that we used to define if a given significant cluster should be tagged as pathogenic or non-pathogenic according to equation 2.

#### Equation 2

Function used to define if a given significant cluster should be tagged as ‘pathogen family’ or ‘non-pathogen family’.
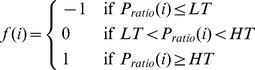
(2)


Let *f* (Equation 2) be the function we use to decide if a given significant cluster should be tagged as pathogenic or non-pathogenic. If the number of sequences from pathogens and non-pathogens is too close in a given cluster (if P*_ratio_* = 0.5 then *f(i)* = 0), the cluster does not have any discriminative value for pathogenicity and is unusable.

Given a protein cluster *i*, it was considered a protein family if the following 3 conditions were satisfied:

- ORG*_i_*≥MinORG

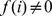

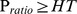
 or 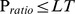

The significance of a protein family depends on its ORG value and its P*_ratio_*. A statistical measure called Z-score (*Z*) was used to take into account the above two values of a family and assess its significance. The estimation of the *Z* values was performed on the set C composed of all the clusters *i* satisfying condition I. Let 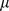 and 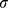 be the average and standard deviation respectively of the P*_ratio_* of the clusters in C. *Z* is a measure representing by how many standard deviations 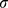 the mean *x* of a sample (a cluster in our case) differs from the mean 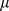 of the population. Given a cluster *i* in C, its mean correspond to its P*_ratio_* and we calculate the *Z* value for *i* as follows:
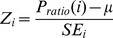
Where *SE* is the standard error of the mean for *i*, and it is:



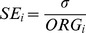



**Figure 1:**
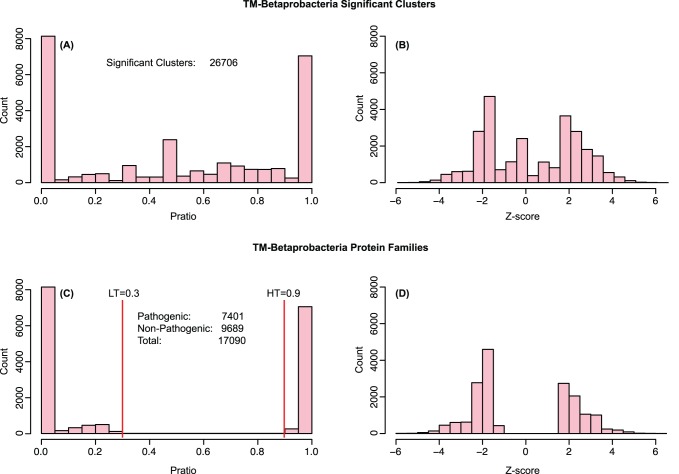
P*_ratio_* and Z-score histograms for TM-Betaproteobacteria model. The model was built setting MinOrg = 2, HT = 0.9 and LT = 0.3. (A) and (B) respectively show the P*_ratio_* and Z-score histograms for the clusters i such that ORG*_i_*≥MinOrg. By this step the original 69,744 clusters are reduced to 26,706. In (A) the bars at the extremes are the count for clusters containing either only genes from pathogenic organisms (right bar) and non-pathogenic ones (left bar), while the small pick in the middle are clusters containing the same number of pathogenic and non-pathogenic organisms, and hence will not be used since they provide no discriminative information about pathogenicity. (C) and (D) show the same histograms for the PFs obtained removing all the significant clusters with P*_ratio_* value between LT and HT. We can see how the amount of non-pathogenic PFs is higher than the pathogenic ones (C). HT and LT can be used to modify the amount of both pathogenic and non-pathogenic PFs, which can be useful in model in which the training-set has an unbalanced amount of pathogenic and non-pathogenic organisms. In (D) the negative Z-scores are associated with non-pathogenic families while the others are for pathogenic PFs.

**Figure 2:**
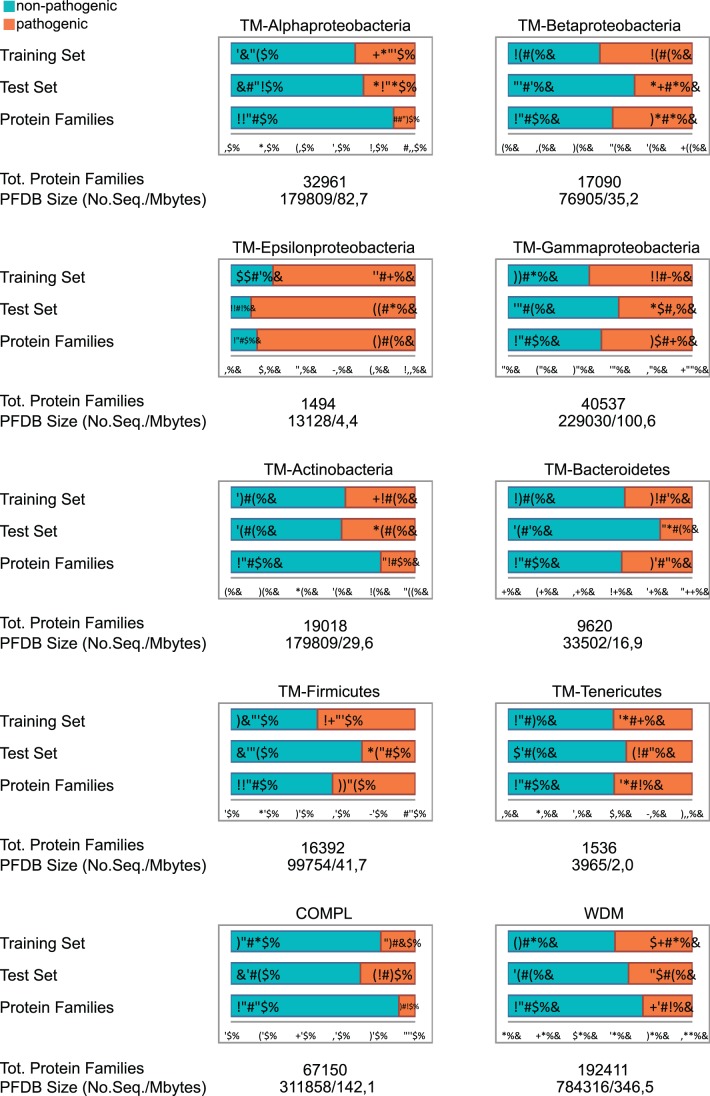
PFDB, training and test-set for each model. Each bar-plot shows the percentage of pathogenic (orange) and non-pathogenic (light-blue) organisms in the training and test-set, and the percentage of pathogenic and non-pathogenic protein families in the PFDB of the model identified by the title of the bar-plot (eg. WMD). Below each horizontal bar-plot the number of protein families composing the PFDB of the model the bar-plot refers to, along with its size in megabytes and the number of sequences, is shown.

To each protein family, a *Z* value was assigned, and these are used in the calculation of the final prediction score as well as a ranking value in the analysis of the protein families. Figure 1 shows the distributions of the P*_ratio_* values and Z-scores for both significant clusters and protein families for the TM-Betaproteobacteria model, while Figure 2 shows for each of the models built the proportion of pathogenic and non-pathogenic families in the PFDB, together with the training-set and test-set for the 10 models built. All the sequences in the PFDB are used to perform the predictions.

### Models Optimisation

The prediction models were verified by 5-fold cross validation. For each of the models, many trials and tests were performed before choosing the MinOrg, LT and HT parameters for the final models. At each CV a parameter called *Zthr*, was further optimised. *Zthr* is the threshold used to decide whether an input organism should be predicted as pathogenic or not, by comparing it to the summation of *Z* values obtained for the matching sequences in the input proteome. The parameters (MinOrg, HT, LT)(Table 1) of the models with the highest MCC in the CV tests were used to create the final models, and the corresponding *Zthr* values will be used as thresholds for the predictions.

### Pathogenicity Prediction

The prediction method takes as input a FASTA file containing the proteins of the organism for which we want to assess the potential pathogenicity. In case the input is a complete or draft genome, initial gene prediction is performed using PRODIGAL [61]. PRODIGAL outputs a set of proteins representing the predicted genes. This is then used as input to our method. Using CD-HIT-2D [60], the input file is compared to the PFDB, and the output will contain all the input sequences that matched sequences in the PFDB, and that are used to compute the final prediction.

The following 4 steps describe the process that leads to the prediction:

- Compare the input proteins to the PFDB
- Filter hits based on the identity threshold (Equation 3)
- Calculate final score summing the *Z* values associated to the matched PFs
- Compare the final score to the model’s *Zthr* threshold and give the final prediction.

From the comparison in step I, we obtain a list of clusters, the representatives of which are sequences belonging to the PFDB, while the non-representative sequences come from the input. Because it is possible that more than one of the input proteins fall inside the same cluster, the sequence with the highest identity percentage with the representative is chosen. [!ht].

#### Equation 3

Calculates the identity threshold to select significant matches that will be used in the final prediction. The calculation is based on statistics on the identity values obtained for all matching query sequences.
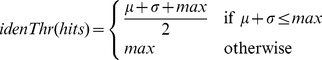
(3)


The list of matches is then filtered based on an identity threshold that is dynamically computed at each prediction using the function idenThr (Equation 3). Let *hits* be a set containing all the percentage identity values for all our matches. Let 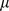 and 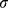 be respectively the average and standard deviation of the percentage identity values in *hits*. Let *max* be the maximum percentage identity obtained for the hits in PFDB. Remembering that, based on the settings of CD-HIT-2D, the minimum identity is 60%. Equation 3 calculates the identity threshold as the middle point between the maximum, and the average increased by one standard deviation, of the identities in *hits*. Selecting all the matches with an identity higher than idenThr(hits), we will obtain a list of hits with a very high identity relatively to the distribution of identities of our hits.

The matches below that threshold will not be used in the final prediction. The process will sometimes greatly reduce the number of matches, but this is in favour of matches with higher identity, making the final prediction more reliable, if compared to the results obtained using a fixed threshold, as we proved by using the paired student’s t-test (results not shown).

In the end we compute the summation of the Z-scores associated with the families matching the input sequences (III). If the sum of the Z-scores is above *Zthr* the input is considered pathogenic, otherwise it is considered non pathogenic (IV).

## Supporting Information

Table S1 **Training and Test organisms.** xlsx file containing the list of organisms in the training and test-set and a table showing the phyla of the organisms in the training-set used to build the COMPL model.
(XLSX)

Table S2 **Extra Escherichia Coli Strains.** xlsx file containing the training-sets used for building *ecoli_boost* and *enterobac_boost* models, including the list of extra *E. coli* strains and a summary of the results in the prediction of *E. coli* and enterobacteriaceae organisms.
(XLSX)

Table S3 **Comparison with other methods.** xlsx file containing a detailed description of the comparison of *PathogenFinder* and the method described in [28].
(XLSX)
