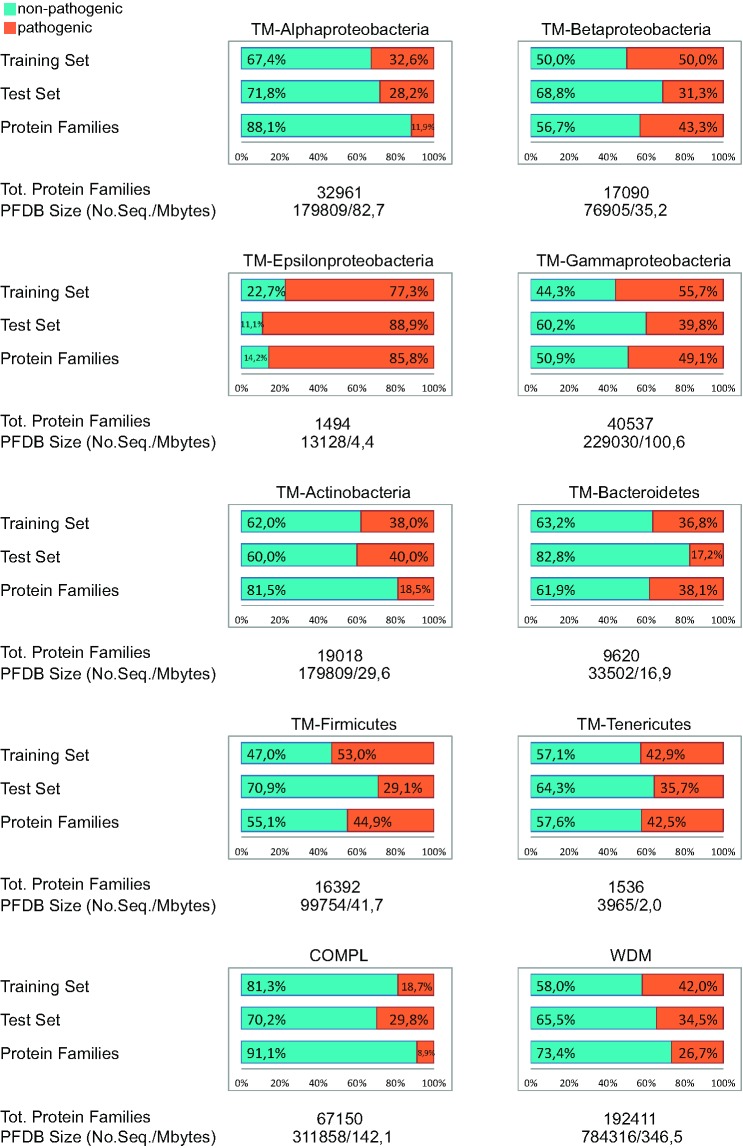# Correction: PathogenFinder - Distinguishing Friend from Foe Using Bacterial Whole Genome Sequence Data

**DOI:** 10.1371/annotation/b84e1af7-c127-45c3-be22-76abd977600f

**Published:** 2013-12-13

**Authors:** Salvatore Cosentino, Mette Voldby Larsen, Frank Møller Aarestrup, Ole Lund

During the production process, many numbers in the figure were converted to symbols. Please see the correct Figure 2 here: 

**Figure:**